# Inflation targeting or exchange rate targeting: Which framework supports the goal of price stability in emerging market economies?

**DOI:** 10.1371/journal.pone.0201798

**Published:** 2018-08-28

**Authors:** Nora Abu Asab, Juan Carlos Cuestas, Alberto Montagnoli

**Affiliations:** 1 Department of Economics, University of Sheffield, Sheffield, United Kingdom; 2 Department of Economics and IEI, Jaume I University, Castellón, Spain; 3 Department of Economics and Finance, Tallinn University of Technology, Tallinn, Estonia; 4 Research Unit, Eesti Pank, Tallinn, Estonia; Universidad de Castilla-La Mancha, SPAIN

## Abstract

The paper investigates and compares the relationship between inflation and inflation uncertainty under inflation targeting and, alternatively, a conventional fixed exchange rate system, for a group of emerging countries. To do so we estimate GARCH in mean models and we find that there is a bi-directional relationship between inflation and inflation uncertainty under the two monetary regimes. It is also found that the fixed exchange rate regime has no impact on average inflation and inflation inertia, while inflation targeting has been successful at lowering both average inflation and inflation persistence.

## Introduction

Since the introduction of fiat money, inflation has become an important problem. This is because inflation may have negative effects on economic dynamics, nominal wages and the intertemporal decisions of market agents. However, the historical evidence on inflation from the last century reveals that the discretionary money standard which are bound to a fixed exchange rate regime and with an independent central bank were less exposed to high inflation [1]. Therefore, the inflationary bias that might be created in the absence of monetary rules has highlighted the need for monetary regimes with a clear monetary constitution.

Nonetheless, there is a general consensus that the social and financial costs attributed to inflation are due mainly to uncertainty about future inflation. The nexus between inflation and inflation uncertainty has gained attention in the literature after the Nobel lecture of [2] pointing out that inflation causes higher inflation uncertainty. The positive relationship between the two variables is theoretically justified by [3], who shows that weak policy makers are more likely to permit inflation during high-inflation episodes, which produces more inflation uncertainty. [4], on the other hand, reveal that inflation uncertainty leads to higher inflation while [5] states that central banks could respond to inflation uncertainty through lowering the money stock; such a stability reaction would make the nexus.

Hence, a large number of studies have been carried out to assess the validity of Friedman-Ball (F-B) and Cukierman and Meltzer (C-M) hypotheses; the literature has grown after the development of the Auto Regressive Conditional Heteroskedasticity (ARCH) and Generalised ARCH (GARCH) techniques, by [6] and [7], respectively. However, only a few studies, e.g., [8], [9], [10], have considered the impact of monetary regimes upon the nexus and focused mainly on inflation targeting (IT) and the monetary union regime. In addition, far too little attention has been paid to cases with soft fixed exchange rates. In some studies e.g., [11] and [12], the role of the regime on the nexus has been ignored altogether.

Therefore, this paper investigates the nexus between inflation and inflation uncertainty in emerging market economies under two monetary anchors: a soft pegged exchange rate (FER) to the US dollar and IT. The aim of this paper is twofold. Firstly, we test the F-B and C-M hypotheses under the two regimes. Secondly, we evaluate the plausible effects of adopting a specific quantitative target on inflation uncertainty. To do this, different GARCH in Mean (GARCH-M) models are constructed to investigate the relationship in two countries with a fixed exchange rate to the US dollar: Jordan and Egypt, and three inflation targeters: South Africa, Brazil and Poland. Note that Egypt has now abandoned the fixed exchange rate system, so the study compares between the time during the fixed system and after abandoning it.

The plan of this paper is as follows; section two provides the literature review. Section three discusses the methodology applied. Section four presents the data and country selection criteria. The results and concluding remarks are provided in sections five and six, respectively.

## Literature review

It is widely believed that the adverse impact of inflation can be limited if inflation is fully anticipated. This notion indicates that the emphasis should be placed on what makes inflation volatile and unpredictable. Hence, the real costs of inflation should be linked to uncontrolled inflation, which affects both the supply and demand sides of the economy, as implied by [13]. Due to unanticipated demand shocks, inflation may increase interest rates, which thereby, impedes investment decisions, reduces the real value of wealth, lowers households’ spending and encourages imported goods and services. In the absence of full wage indexation, inflation can have an adverse impact on real incomes. On the supply side, inflation distorts the management of economic resources, and adds additional costs to acquire accurate information, which could potentially be costly, particularly for small firms. The effect of inflation is also tremendously important in financial markets. This is because it influences both ex-ante and ex-post financial decisions. In fact, as high inflation raises uncertainty about future inflation, market agents tend to seek higher returns or higher nominal interest rates, while simultaneously trying to hedge their wealth by spending more resources on forecasting inflation. The forecasts, however, might mispredict the real inflation, leading to inferior redistributive impacts on agents’ real balances [14].

Some policy makers and economists suggest that the costs of a predictable low and moderate inflation rate are acceptable and supported by the economic theory. However, [15] shows that an anticipated rate of steady inflation as implied by accelerationists would be ideal to wind down the social and redistributive costs of inflation, but such a steady inflation level is difficult to achieve due to inflation expectations, which hinge substantially upon the type of government in power and the trade-off between employment and inflation. Consequently, he points out that the acceptance of moderate and steady inflation would trigger higher inflation expectations, which eventually leads to a higher inflation rate. Furthermore, he hypothesises, by analysing the inflation behaviour for different OECD countries, that high inflation may lead to higher inflation variability, and that high inflation countries experienced higher inflation variability. However, the link between inflation and its uncertainty has gained much interest after the Nobel lecture of [2]. Friedman states that the relationship between unemployment and nominal wage changes is not stable owning to inflation uncertainty, which increases with the level of inflation. He further argued that inflation-inflation uncertainty leads to lower output growth; however, this impact is unsettled in the literature and depends on whether money is considered to be neutral; see e.g., [16], [17], [18]. Recent studies have shown that the link exists in practice, but the effect of inflation on economic growth comes through inflation uncertainty, see [8], [19]. [20], on the other hand, finds that output growth is not affected by inflation uncertainty. [3] supports the hypothesis suggested by Friedman that high inflation leads to higher inflation uncertainty. He bases his argument on a monetary policy-time inconsistency game theoretical model of [21], where market agents are uncertain about the type of government in power. As long as inflation remains low, both weak and strong types of policy makers will keep it low. Yet, the dilemma appears when inflation could be permitted by the weak policy maker, during high inflation episodes, and thereby, inflation uncertainty will tend to be high when inflation is high. On the other hand, on the basis of the same time inconsistency model, [4] argue that, as central bankers are motivated to create surprise inflation to stimulate the economic activity, an increase in inflation variability raises the inflation rate. Policy makers may increase an optimal inflation rate to benefit from low unemployment, see [22], or to lower the public debt, see [23] for further detail. In other words, for Friedman and Ball, higher inflation creates higher inflation uncertainty, while for Cukieman-Meltzer, the link goes in the other direction, that is, inflation uncertainty increases inflation. [5], however, shows that a negative link could be an indicator of the stabilising effects of the monetary framework conducted by central banks.

A large number of empirical works have attempted to examine the nexus between inflation and inflation uncertainty using different measures of inflation uncertainty. [24] and [25] study the nexus for Latin American countries. While the former suggests that market agents update their inflation expectations adaptively, the latter bases their investigation on rationality of agents, in which responses to changes in inflation happen over shorter horizons. [25] find that, by regressing the absolute value of expected inflation errors on realised inflation, higher inflation hinders the predictability of prices changes. However, their results are weaker compared to [24], in which the latter uses the difference between expected and realised inflation as a measure of unpredicted inflation. Nevertheless, as it is not possible in reality to determine the inflation expectations formation process employed by market agents, such estimations for the nexus could be misleading [26]. Hence, other studies have attempted to investigate the relationship by utilising inflation forecasts gathered from surveys of inflation expectations. [27] use the mean square of inflation forecast error to proxy for inflation uncertainty, whereas [26] adopt two other measures of inflation unpredictability along the mean squared error: the absolute forecast error of [24] and the squared forecast error of [28]. Both studies confirm the existence of a positive relationship between inflation and its unpredictability. [14] employs the standard deviation of survey participants’ inflation forecasts and proves that uncertainty increases with inflation. See also [29] and [30].

Although the survey-based measures were believed to be good proxies for inflation uncertainty, such measures were unable to distinguish between transitory and permanent shocks to inflation, where the latter has a much stronger effect on the intertemporal decision making of individuals and businesses. Thus, [31] claim that the effect of control errors lasts temporarily and decays over short periods, whereas inflation has a severe effect on uncertainty at longer horizons, where permanent shocks dominate. [6] was the first to measure inflation uncertainty as the conditional variance of inflation to study the relationship between inflation and uncertainty in the United States. In fact, the introduction of Autoregressive Conditional Heteroscedasicity (ARCH) and General Autoregressive Conditional Heteroscedasicity (GARCH) approaches by [6] and [7], respectively, encouraged a large number of recent empirical studies examining the link between inflation and inflation uncertainty.

[32] constructs a model which allows for the changes in the structure of inflation to affect inflation uncertainty. This is performed by incorporating the different aspects of inflation uncertainty through the Kalman filter: the conditional variance of inflation, the conditional variance of expected inflation and the conditional variance of steady-state inflation. He applies the model to the US during the period 1960:01-1988:06 and concludes that inflation raises inflation uncertainty. [33] examine the relationship utilising a GARCH model to produce a proxy for inflation uncertainty in the G7 countries. Their findings from Granger causality tests suggest that the nexus is positive as implied by F-B, but little evidence is found in favour of the C-M hypothesis. [34] and [9], who apply GARCH and GARCH-M, respectively, find evidence that the F-B hypothesis, for different examined periods, held true for the UK. In fact, most studies have used either GARCH or GARCH-M to investigate the relationship between inflation and uncertainty, in different countries, reporting mixed results concerning the causality between inflation and uncertainty; whether it goes from inflation to uncertainty or the opposite; others even find the link to be bi-directional. [35] find evidence for the Friedman hypothesis in the US and the UK, while the results for Japan show that uncertainty affected the level of inflation as suggested by C-M.

One drawback of the GARCH model is that it is unable by its construction to capture asymmetric responses to positive and negative shocks to inflation. Hence a new family of asymmetric GARCH models has evolved to consider the fact that bad news in financial markets has deeper effects than good news [33]. Subsequently, many studies have constructed different models of asymmetric GARCH. [36] examine the nexus, for five Asian countries: Indonesia, Malaysia, Philippines, Singapore and Thailand, using EGARCH, and find evidence in favour of both the F-B and C-M hypotheses. They note that inflation is a threat, even in low inflation countries, as it leads to increased uncertainty. In a similar vein, [37] use the same model to generate a measure for inflation uncertainty in Iran and find that inflation granger causes inflation uncertainty. The same conclusion is reached by [38] and [11], who study the relationship for Pakistan and a number of emerging market economies and the G7, respectively. However, [11] show that positive shocks to inflation have more powerful impacts on inflation uncertainty than negative ones, especially in Latin American countries, while [38] find that the opposite is true for Pakistan. [39] also found that positive inflation shocks have stronger effects on uncertainty. [40] employs EGARCH-M to investigate the link between inflation, inflation uncertainty and output growth in Japan. His findings are in the line with [2], that is, high inflation leads to higher inflation uncertainty and lower productivity. He also finds that negative shocks increase inflation uncertainty more than positive shocks. [41] find that the relationship between inflation and uncertainty, in five of the six European countries considered in their analysis, over the period 1960-1999, is consistent with the Friedman hypothesis.

A few studies have applied the Markov regime switching AutoRegressive Heteroscedastic (SWARCH) model to take account of shifts in the monetary regime in investigating the relationship between inflation and inflation uncertainty. For Peru, [42] show that the shift to adopt inflation targeting is characterised by less inflation volatility, and that higher inflation raises inflation uncertainty. [43] utilise the SWARCH model to study the impact of inflation targeting on inflation uncertainty for a number of developed and emerging market countries. According to their findings, IT lowers the variance of inflation in most of the countries examined; however, this positive advantage increases with high level of central bank transparency and the institutional arrangements.

In fact, the adoption of inflation targeting across many developed and developing central banks has increased the appetite to discover the benefits of the new framework. [34] suggests that the announcement of an explicit inflation target has a prominent effect on lowering inflation persistence and uncertainty at long horizons. [44] also supports the Friedman hypothesis for the United States as the nexus is found to be positively correlated during high inflation periods. Likewise, [43] find that the relationship between inflation and inflation uncertainty is positive and inflation variance has decreased after IT was adopted in most inflation targeters investigated in his study. Similar findings were obtained by [9] for the UK. [45] also point out that IT is an optimal anchor for the UK, however, they find that the relationship between inflation and uncertainty has become negative after inflation targeting. Furthermore, [46] [45] claim that adopting IT in New Zealand and Australia granted monetary authorities more flexibility in setting the nominal interest rates. See also [47] on the relationship between inflation uncertainty and interest rates for five inflation targeters, and [48] for the effect of inflation targeting on interest rates. Other studies; [49], [50], [10], show that adopting the Euro played a major positive role in affecting the nexus between inflation and uncertainty. The only study which compared between two different monetary regimes: currency boards and inflation targeting, for Eastern European countries, is [51], who apply the EGARCH model and find support for the Friedman hypothesis. However, the study fails to determine which monetary anchor worked better at reducing inflation uncertainty.

## Methodology

GARCH-type models have been widely employed to investigate the relationship between inflation and inflation uncertainty, as they provide a time-varying measure for volatility. Nevertheless, a standard GARCH model does not allow examining the effects of inflation and inflation uncertainty simultaneously. Hence, the previous statistical technique to study the direction of the nexus, conducted by some studies, was a two-stage approach, where the conditional variance of inflation is estimated in the first stage before performing the Granger causality tests to determine the direction of causality.

However, the GARCH in mean model, developed by [52], permits inflation to be specified by inflation uncertainty. Therefore, to investigate the relationship simultaneously, the conditional variance of inflation is allowed to be influenced by the mean, and the inflation rate to be determined by the conditional variance. GARCH model estimates a time-varying variance of residuals which acts as a proxy for unexpected inflation volatility [11]. The GARCH regression model is built on an autoregressive moving average of a known variable, where the conditional variance is a linear function of its past values and past squared shocks. This model has an ARMA construction which makes the model soluble; however, given its quadratic specification, the model equalises the effect of positive and negative innovations [53]. Inserting a one-period lagged inflation in the variance equation allows examination of the hypothesis of [2] and [3]. In our study, as explained in detail in the results section, the mean equation is built on AR specifications rather than ARMA process, so the lagged error terms are excluded. These specifications can be represented as follows:

The mean equation:

$$\begin{array}{c} \pi_{t}=\alpha_{0}+\sum_{t=1}^{p}\alpha_{i}\pi_{t-i}+\sum_{j=1}^{q}\beta j\varepsilon_{t-j}+\delta \sqrt{h_{t}}+\varepsilon_{t} \end{array}$$ (1)

The variance equation:

$$\begin{array}{c} \pi_{t}=\phi +\sum_{t=1}^{p}\alpha_{i}\varepsilon_{t-1}^{2}+\sum_{j=1}^{q}\beta jh_{t-j}+\lambda Z_{t-1} \end{array}$$ (2)

Where *π*_*t*−*i*_ is the lagged inflation rate; *ε*_*t*−*j*_ is the lagged errors and *ε*_*t*_ is the error term, which has conditional and unconditional mean of zero and conditional variance, *h*_*t*_, given by Eq (2). The conditional variance is determined by the lagged squared residuals, the lagged conditional variance and Z_*t*−1_, which includes only lags of inflation. Stationarity restrictions of the model entail that *α*_*i*_ and *βj*, the non-negative parameters, must be less that unity. If the sum of the parameters is equal to one, the conditional variance must be modelled by Integrated GARCH [54].

If *δ* in the mean equation is significantly positive, higher inflation uncertainty generates higher inflation, as argued by C-M. On the other hand, when the coefficient is significantly negative, the Holland hypothesis of monetary policy stabilising effect holds true. λ in Eq (2) determines the effect of inflation on inflation uncertainty. Obtaining a positive and significant coefficient indicates that inflation uncertainty increases with inflation, as suggested by F-B.

Nevertheless, the conditional variance of inflation estimated by GARCH-M is formed to consider only the magnitude of inflation shocks, $\varepsilon_{t-1}^{2}$, and thereby the sign of innovations is ignored by the model construction. Hence, to account for possible asymmetric responses to positive and negative inflation shocks, an asymmetric GARCH model, i.e., Exponential GARCH, is used.

The conditional variance in the EGARCH model, put forward by [55], is set in a logarithmic form, which does not require imposing artificial non-negativity constraints on the parameters to ensure a positive variance. The model representation can be seen as follows:

$$\begin{array}{c} \log h_{t}^{2}=\varphi +\beta_{1}\left[\right|\frac{\varepsilon_{t-1}}{h_{t-1}}\left|\right]+\beta_{2}\left[\frac{\varepsilon_{t-1}}{h_{t-1}}\right]+\beta_{3}\log h_{t-1}^{2}+\lambda Z_{t-1} \end{array}$$ (3)

In this case, an asymmetric response to inflation shocks exists if *β*_2_≠0. A significantly positive *β*_2_ implies that the inflation uncertainty increases more when the economy is hit by a positive shock, i.e., *ε*_*t*−1_>0, than by a negative inflation shock, i.e., *ε*_*t*−1_<0 [40].

Nevertheless, as policy makers are more concerned with the long-run impact of inflation uncertainty, and more importantly the impact of a monetary anchor on reducing inflation uncertainty in the long-run, we utilise the Component GARCH (CGARCH) model, developed by [56]. This model separates the long-run from short-run components of inflation uncertainty by allowing the mean of the conditional variance to vary around a time varying level, *φ*.

$$\begin{array}{c} h_{t}=\varphi_{t}+\alpha_{1}\left(e_{t-1}^{2}-\varphi_{t-1}\right)+\beta_{1}\left(h_{t-1}+\varphi_{t-1}\right) \end{array}$$ (4)

$$\begin{array}{c} h_{t}=\varphi +\rho \varphi_{t-1}+\mu \left(e_{t-1}^{2}-h_{t-1}\right)+\lambda Z_{t-1} \end{array}$$ (5)

Eq (4) represents the transitory component, which approaches zero with the power of *α*_1_+ *β*_1_. *ρ* in the long run component, shown in Eq (5), is usually close to one, as the time varying trend converges to the mean very slowly. If, 1>*ρ*>*α*_1_+*β*_1_, the short run component of inflation uncertainty will die out more rapidly than the trend. This indicates that the forecasts of the conditional variance will depend essentially upon the trend [9].

## Data and developing countries selection criteria

We select inflation targeters from three different continents that had experienced an economic and/or political challenge before shifting to IT. The exchange rate targeting countries represent cases which also experienced a shift in monetary regime during the study period and suffered from political and economic pressures, criteria which do not apply to most exchange rate peggers, e.g., Gulf States. This selection allows us to highlight how the economies of the examined countries have benefited from the regime under investigation. The paper implicitly compares the impact of monetary policy independence on inflation and inflation uncertainty, where inflation targeting enjoys more monetary policy flexibility compared to the soft pegged exchange rate system.

Monthly data on the Consumer Price Index (CPI) for the period from 1980:01 to 2014:06 are extracted from the International Financial Statistics of the International Monetary Fund for the sample countries, http://data.imf.org/regular.aspx?key=61545861. The monetary regime shift experienced by all the countries allows us to highlight the benefits of the examined regime in terms of inflation and inflation uncertainty.

Inflation is computed as *π*_*t*_ = log cpi_*t*_ − log cpi_*t*−1_. The inflation series is then adjusted to remove the seasonality by executing the Census Bureau’s X12 in an additive default mode.

## Results

The conditional mean of inflation is specified by constructing several ARMA models. Given that inflation is seasonally adjusted, it is found that including only the autoregressive terms yields the best model specifications, which is commonly the case in modelling inflation in the empirical literature [9]. For each country, we begin by incorporating up to twelve AR specifications to capture the persistence of the data. The length of AR components is shortened on the basis of Akaike and Schwartz information criteria and by ensuring that all autocorrelation coefficients up to twelve lags fall inside the non-rejection region, which is also confirmed by the Q-statistics of [57]. Accordingly, the selected AR process forms the following benchmark-mean specifications:

Jordan:

$$\begin{array}{c} \pi_{t}=\gamma_{0}^{JO}+\gamma_{1}^{JO}\pi_{t-2}+\gamma_{2}^{JO}\pi_{t-5}+\gamma_{3}^{JO}\pi_{t-9}+\gamma_{4}^{JO}\pi_{t-12}+u_{t} \end{array}$$ (6)

Egypt:

$$\begin{array}{c} \pi_{t}=\gamma_{0}^{EG}+\gamma_{1}^{EG}\pi_{t-1}+\gamma_{2}^{EG}\pi_{t-9}+\gamma_{3}^{EG}\pi_{t-12}+u_{t} \end{array}$$ (7)

South Africa:

$$\begin{array}{c} \begin{array}{c} \pi_{t}=\gamma_{0}^{SA}+\gamma_{1}^{SA}\pi_{t-1}+\gamma_{2}^{SA}\pi_{t-2}+\gamma_{3}^{SA}\pi_{t-3}+\gamma_{4}^{SA}\pi_{t-7}\\ +\gamma_{5}^{SA}\pi_{t-8}+\gamma_{6}^{SA}\pi_{t-11}+\gamma_{7}^{SA}\pi_{t-12}+u_{t} \end{array} \end{array}$$ (8)

Brazil:

$$\begin{array}{c} \pi_{t}=\gamma_{0}^{BR}+\gamma_{1}^{BR}\pi_{t-1}+\gamma_{2}^{BR}\pi_{t-2}+\gamma_{3}^{BR}\pi_{t-8}+u_{t} \end{array}$$ (9)

Poland:

$$\begin{array}{c} \pi_{t}=\gamma_{0}^{PO}+\gamma_{1}^{PO}\pi_{t-1}+\gamma_{2}^{PO}\pi_{t-2}+\gamma_{3}^{PO}\pi_{t-5}+\gamma_{4}^{PO}\pi_{t-9}+\gamma_{5}^{PO}\pi_{t-11}+u_{t} \end{array}$$ (10)

For each country, we split the inflation series between the time before and after adopting the monetary regime of interest. The preliminary evidence from the OLS regression of the benchmark models, shown in Tables 1 to 5, suggests that IT has been successful at reducing the volatility of inflation as the ARCH effect turned insignificant after adopting IT. Interestingly, for Egypt, inflation volatility became lower not during the fixed exchange rate system but after becoming an inflation targeter, while for Jordan, the impact of exchange rate targeting is unclear as the volatility for the period after exchange rate targeting is found at high lags.

**Table 1:** OLS estimates of inflation conditional mean for Jordan.

coefficient	full sample 1981:02-2014:06	pre-target 1981:02-1995:09	post-target 1995:10-2014:06
$\gamma_{0}^{JO}$	0.003***	0.004***	0.003***
$\gamma_{2}^{JO}$	0.117**	0.154**	0.002
$\gamma_{5}^{JO}$	0.173***	0.259***	-0.031
$\gamma_{9}^{JO}$	0.099**	0.125*	0.019
$\gamma_{12}^{JO}$	-0.100**	-0.082	-0.174***
ARCH(1)	8.20***	5.68**	0.218
ARCH(2)	17.36***	13.70***	0.217
ARCH(12)	26.66***	20.25*	21.13**

Note: ARCH(1), ARCH(2) and ARCH(12) are ARCH test at 1^*st*^, 2^*nd*^ and 12^*th*^ lag, respectively. Rejection of the null hypothesis at the 10%, 5% and 1% significance level are given by the symbols *, ** and ***, respectively.

**Table 2:** OLS estimates of inflation conditional mean for Egypt.

coefficient	full sample 1981:02-2014:06	during-target 1981:02-2002:12	opting out 2003:01-2014:06
$\gamma_{0}^{EG}$	0.008***	0.009***	0.007***
$\gamma_{1}^{EG}$	-0.155***	-0.199***	0.380***
$\gamma_{9}^{EG}$	0.124***	0.142**	-0.098
$\gamma_{12}^{EG}$	-0.144***	-0.144***	-0.084
ARCH(1)	65.02***	38.68***	0.162
ARCH(2)	71.46***	42.85***	0.249
ARCH(12)	104.90***	62.33***	8.85

Note: ARCH(1), ARCH(2) and ARCH(12) are ARCH test at 1^*st*^, 2^*nd*^ and 12^*th*^ lag, respectively. Rejection of the null hypothesis at the 5% and 1% significance level are given by the symbols ** and ***, respectively.

**Table 3:** OLS estimates of inflation conditional mean for Brazil.

coefficient	full sample 1981:02-2014:06	pre-target 1981:02-1999:06	post-target 1999:07-2014:06
$\gamma_{0}^{BR}$	0.063**	0.110***	0.005***
$\gamma_{1}^{BR}$	0.462***	0.447***	0.741***
$\gamma_{2}^{BR}$	0.367***	0.358***	-0.062
$\gamma_{8}^{BR}$	0.085**	0.065	0.035
ARCH(1)	25.58***	13.08***	1.29
ARCH(2)	25.69***	13.13***	4.36
ARCH(12)	29.16***	14.63	8.3

Note: ARCH(1), ARCH(2) and ARCH(12) are ARCH test at 1^*st*^, 2^*nd*^ and 12^*th*^ lag, respectively. Rejection of the null hypothesis at the 5% and 1% significance level are given by the symbols ** and ***, respectively.

**Table 4:** OLS estimates of inflation conditional mean for South Africa.

coefficient	full sample 1981:02-2014:06	pre-target 1981:02-2000:01	post-target 2000:02-2014:06
$\gamma_{0}^{SA}$	0.007***	0.008***	0.004***
$\gamma_{1}^{SA}$	0.231***	0.139**	0.397***
$\gamma_{2}^{SA}$	0.213***	0.228***	0.048
$\gamma_{3}^{SA}$	0.134***	0.108*	0.176
$\gamma_{7}^{SA}$	0.131***	0.173***	-0.032
$\gamma_{8}^{SA}$	0.100***	0.106*	0.07
$\gamma_{11}^{SA}$	0.106***	0.114**	0.003
$\gamma_{12}^{SA}$	-0.124***	-0.089	-0.214***
ARCH(1)	34.47***	12.82***	0.27
ARCH(2)	34.90***	13.16***	1.77
ARCH(12)	56.67***	26.60***	18.12

Note: ARCH(1), ARCH(2) and ARCH(12) are ARCH test at 1^*st*^, 2^*nd*^ and 12^*th*^ lag, respectively. Rejection of the null hypothesis at the 10%, 5% and 1% significance level are given by the symbols *, ** and ***, respectively.

**Table 5:** OLS estimates of inflation conditional mean for Poland.

coefficient	full sample 1981:02-2014:06	pre-target 1981:02-1998:08	post-target 1998:09-2014:06
$\gamma_{0}^{PO}$	0.008***	0.013***	0.003***
$\gamma_{1}^{PO}$	0.218***	0.181***	0.294***
$\gamma_{2}^{PO}$	0.232***	0.221***	0.136*
$\gamma_{5}^{PO}$	0.138***	0.118*	0.128*
$\gamma_{9}^{PO}$	0.114**	0.099	0.093
$\gamma_{11}^{PO}$	0.181***	0.177***	0.113
ARCH(1)	19.96***	8.34***	0.02
ARCH(2)	20.61***	9.79***	0.03
ARCH(12)	36.78***	18.46	1.4

Note: ARCH(1), ARCH(2) and ARCH(12) are ARCH test at 1^*st*^, 2^*nd*^ and 12^*th*^ lag, respectively. Rejection of the null hypothesis at the 10%, 5% and 1% significance level are given by the symbols *, ** and ***, respectively.

In order to examine the simultaneous relationship between inflation and inflation uncertainty, we incorporate the standard deviation in the mean equation as a volatility measure and augment the variance equation with lagged inflation. Inserting S.D in the mean equation is used by [58] and [9]. Furthermore, to model the impact of the monetary regime on inflation dynamics, slope dummies are plugged in to the conditional mean equations, in which the dummy takes the value of one when the examined monetary regime is in effect, and zero otherwise. We first attempt to introduce the regime slope dummies via different lags, but only two interactive dummies are selected to interact with their corresponding inflation lags based upon a significant improvement in the fit of the model. We also employ a constant regime dummy in the mean equation for the cases where doing so is found to substantially improve the overall statistical performance. In addition, we account for political circumstances in some countries. For the two exchange rate targeters, a dummy variable is added to the mean equation to capture the impact of the Arab Spring on average inflation, in which the dummy is assigned one for the period from 2011:01 onwards. The unrest and tensions across the Middle East spread to Jordan and had negative effects on the economy. For instance, the pipelines that carried gas from Egypt to Jordan were targeted and bombed several times during the uprising, resulting in oil supply shortage. As a consequence, Jordan was forced to deal with Israel to import gas, as Israel has become a major gas exporter in the region. However, the pact to deal with Israel triggered more domestic opposition and increased the external debt; see the Daily Mail on 11^*th*^ December 2014 for more details. The Arab Spring countries are still, at the time of writing this, being affected by the adverse consequences of the social unrest. For South Africa, a constant dummy is included to consider the effect of apartheid on inflation, which takes the value of one for the period before January 1995, and zero for the months thereafter. Hence, the augmented mean equations can be represented as follows:

Jordan:

$$\begin{array}{c} \pi_{t}=\left(\delta_{1}^{JO}+\delta_{3}^{JO}\right)\pi_{t-2}+\delta_{4}^{JO}\pi_{t-5}+\delta_{5}^{JO}\pi_{t-9}+\left(\gamma_{2}^{JO}+\delta_{6}^{JO}\right)\pi_{t-12}+u_{t} \end{array}$$ (11)

Egypt:

$$\begin{array}{c} \pi_{t}=\left(\delta_{1}^{EG}+\delta_{3}^{EG}\right)\pi_{t-1}+\delta_{4}^{EG}\pi_{t-9}+\left(\gamma_{2}^{EG}+\delta_{5}^{EG}\right)\pi_{t-12}+u_{t} \end{array}$$ (12)

South Africa:

$$\begin{array}{c} \begin{array}{c} \pi_{t}=\left(\delta_{1}^{SA}+\delta_{3}^{SA}\right)\pi_{t-1}+\delta_{4}^{SA}\pi_{t-2}+\delta_{5}^{SA}\pi t-3+\delta_{6}^{SA}\pi_{t-7}\\ +\delta_{7}^{SA}\pi_{t-8}+\delta_{8}^{SA}\pi_{t-11}+\left(\delta_{2}^{SA}+\delta_{9}^{SA}\right)\pi_{t-12}+u_{t} \end{array} \end{array}$$ (13)

Brazil:

$$\begin{array}{c} \pi_{t}=\left(\delta_{1}^{BR}+\delta_{3}^{BR}\right)\pi_{t-1}+\delta_{4}^{BR}\pi_{t-2}+\left(\delta_{2}^{BR}+\delta_{5}^{BR}\right)\pi_{t-8}+u_{t} \end{array}$$ (14)

Poland:

$$\begin{array}{c} \begin{array}{c} \pi_{t}=\left(\delta_{1}^{PO}+\delta_{3}^{PO}\right)\pi_{t-1}+\delta_{4}^{PO}\pi_{t-2}+(\delta_{2}^{PO}\\ +\delta_{5}^{PO})\pi_{t-5}+\delta_{6}^{PO}\pi_{t-9}+\delta_{7}^{PO}\pi_{t-11}+u_{t} \end{array} \end{array}$$ (15)

A joint significance of the interactive regime dummies is confirmed by a Wald test of *δ*_1_ = *δ*_2_ = 0. For each country, Chi-square statistics reject the hypothesis that the dummies are zero at 1% level of significance, as shown in the last row of Tables 6 to 12. The effect of the monetary regime on inflation inertia is reflected by the sum of the coefficients of the regime interactive dummy and that of their corresponding lags. A negative slope dummy indicates that inflation persistence has declined after adopting the examined regime. Note that, as stated by [9], the effect of inflation regime on the inflation persistence is preferred to be analysed in the context of the Kalman filter. The results, reported in Tables 6 to 12, imply that IT has been successful at reducing inflation persistence at a high lag order, as the coefficients of the second interactive dummies, i.e., *δ*_2_, appear with a negative sign. A large body of literature has pointed out that IT helps countries with their disinflationary efforts, stabilises inflationary expectations and enhances the monetary authority’s accountability and transparency, see for example [59], [60], [61], [62], [63], [64], [59]. This, however, does not apply to Poland, where all the slope dummies are non-negative, but its regime constant dummy, D_*t*_, plugged in to the mean equation, shows that the mean of inflation was reduced by IT; this also applies to all the ITers in the sample. We did not report the results with a constant dummy for the other ITers as the results were found to be better, in terms of diagnostics, without adding the regime constant. However, the constant dummy appeared with a negative sign for all the IT cases. We also attempted to incorporate the regime dummy variable in the variance equations, but the dummy was insignificant for all the cases. For South Africa, the years of apartheid were associated with higher average inflation, as the constant dummy, APART, presented in Table 10, is significantly positive under all GARCH models. For Egypt and Jordan, the Arab Spring dummy is found to be insignificant as a constant, but its slope, POL, reported in Tables 6 and 8, has a positive and significant influence on the trend at 1%. Note that the ARCH effect exists in the estimated EGARCH-M model for Egypt, indicating that the model is not well specified. In general, for both countries, all GARCH-M models showed better results when the POL-slope dummy and the regime constant dummy were dropped from the mean equation, see Tables 6 and 8. Unlike the ITers, the inflation mean of the FER targeters is not affected by the fixed exchange rate regime. The dummy appears insignificant for Jordan and positive for Egypt. Nonetheless, the FER system appears to be influential in lowering the inflation inertia in Egypt and Jordan at the first inflation lag.

**Table 6:** GARCH-M models for Jordan (with dummies).

Coefficients	GARCH-M	EGARCH-M
**Conditional mean**		
D_*t*_	-3.97E-04	-3.20E-04
Pol	0.176***	0.187***
$\delta_{1}^{JO}$	-0.148***	-0.125***
$\delta_{2}^{JO}$	0.091***	0.077***
$\delta_{3}^{JO}$	0.116***	0.081***
$\delta_{4}^{JO}$	0.061**	0.047**
$\delta_{5}^{JO}$	0.036	0.019
$\delta_{6}^{JO}$	-0.207***	-0.208***
*δ*	0.328***	0.337***
**conditional variance**		
*ϕ*	7.47E-06**	-1.590***
*α*_1_	0.027	
*β*_1_	0.810***	0.127
*β*_2_		0.142
λ_0_	0.003***	19.19
λ_1_	-0.002***	-25.67***
**diagnostic statistics**		
	*Q*(1) = 2.15	*Q*(1) = 1.27
	*Q*(12) = 13.22	*Q*(12) = 13.70
	*Q*^2^(12) = 14.05	*Q*^2^(4) = 13.94
	TR^2^(12) = 14.6	TR^2^(12) = 14.28
Wald test	20.7***	

Note: *δ* tests the validity of [4] hypothesis, where a positive *δ* indicates that inflation uncertainty increases inflation. λ_0_ is the one-period lagged inflation and tests the validity of [2]–[3] hypothesis, where a positive λ_0_ means that inflation raises inflation uncertainty. D_*t*_ is the constant-monetary regime, i.e., fixed exchange rate system, dummy variable. Pol is the slope dummy that acts for the effect of Arab Spring on average inflation. Wald test examines the significance of the interactive regime dummy, i.e., λ_1_ = λ_2_ = 0. Rejection of the null hypothesis at the 5% and 1% significance level are given by the symbols ** and ***, respectively.

**Table 7:** GARCH-M models for Jordan (without dummies).

Coefficients	GARCH-M	EGARCH-M	CGARCH-M
**Conditional mean**			
$\delta_{1}^{JO}$	-0.143***	-0.131***	-0.139***
$\delta_{2}^{JO}$	0.063*	0.026	0.051
$\delta_{3}^{JO}$	0.095**	0.085***	0.117***
$\delta_{4}^{JO}$	0.059**	0.113***	0.063**
$\delta_{5}^{JO}$	0.058**	0.045***	0.044*
$\delta_{6}^{JO}$	-0.194***	-0.153***	-0.179***
*δ*	0.361***	0.341***	0.338***
**conditional variance**			
*ϕ*	7.95E-06**	-2.493**	4.74E-05
*α*_1_	0.034		0.005
*β*_1_	0.785***	0.121	0.764
*β*_2_		0.052	
λ_0_	0.003***	28.851*	0.003***
λ_1_	-0.002**	-24.709*	-0.003**
*τ*			
*μ*			0.025
*ρ*			0.823***
**diagnostic statistics**			
	*Q*(1) = 2.21	*Q*(1) = 2.34	*Q*(1) = 2.27
	*Q*(12) = 12.23	*Q*(12) = 17.85	*Q*(12) = 13.01
	*Q*^2^(12) = 14.71	*Q*^2^(12) = 21.18	*Q*^2^(12)15.26
	TR^2^(12) = 15.22	TR^2^(12) = 21.05	TR^2^(12) = 15.82
Wald test	10.58***		

Note: *δ* tests the validity of [4] hypothesis, where a positive *δ* indicates that inflation uncertainty increases inflation. λ_0_ is the one-period lagged inflation and tests the validity of [2]–[3] hypothesis, where a positive λ_0_ means that inflation raises inflation uncertainty. Wald test examines the significance of the interactive regime dummy, i.e., λ_1_ = λ_2_ = 0. Rejection of the null hypothesis at the 10%, 5% and 1% significance level are given by the symbols *, ** and ***, respectively.

**Table 8:** GARCH-M models for Egypt (with dummies).

Coefficients	GARCH-M	EGARCH-M
**Conditional mean**		
D_*t*_	0.002***	0.0003*
Pol	0.999***	0.999***
$\delta_{1}^{EG}$	-0.223***	-0.131***
$\delta_{2}^{EG}$	-0.008	-0.137***
$\delta_{3}^{EG}$	0.151***	-0.008***
$\delta_{4}^{EG}$	-0.033	-0.001***
$\delta_{5}^{EG}$	-0.083	0.002***
*δ*	0.621***	0.679***
**conditional variance**		
*ϕ*	9.29E-09	0.170***
*α*_1_	0.399***	
*β*_1_	0.664***	0.265***
*β*_2_		0.956***
λ_0_	3.65E-06	-59.505***
λ_1_	4.43E-04*	30.83***
**diagnostic statistics**		
	*Q*(1) = 3.29	*Q*(1) = 2.78
	*Q*(12) = 40.10	*Q*(12) = 14.54
	*Q*^2^(12) = 4.43	*Q*^2^(12) = 24.69
	TR^2^(12) = 4.16	TR^2^(12) = 26.19**
Wald test	16.39***	

Note: *δ* tests the validity of [4] hypothesis, where a positive *δ* indicates that inflation uncertainty increases inflation. λ_0_ is the one-period lagged inflation and tests the validity of [2]–[3] hypothesis, where a positive λ_0_ means that inflation raises inflation uncertainty. D_*t*_ is the constant-monetary regime, i.e., fixed exchange rate system, dummy variable. Pol is the slope dummy that acts for the effect of Arab Spring on average inflation. Wald test examines the significance of the interactive regime dummy, i.e., λ_1_ = λ_2_ = 0. Rejection of the null hypothesis at the 10%, 5% and 1% significance level are given by the symbols *, ** and ***, respectively.

**Table 9:** GARCH-M models for Egypt (without dummies).

Coefficients	GARCH-M	EGARCH-M	CGARCH-M
**Conditional mean**			
$\delta_{1}^{EG}$	-0.282***	-0.263***	-0.161**
$\delta_{2}^{EG}$	-0.068	-0.047	-0.034
$\delta_{3}^{EG}$	0.189***	0.204***	0.138***
$\delta_{4}^{EG}$	-0.105***	-0.071**	-0.05
$\delta_{5}^{EG}$	-0.159***	-0.166***	-0.151***
*δ*^*EG*^	0.986***	0.987***	0.984***
**conditional variance**			
*ϕ*	3.18E-07	-0.062	9.88E-05**
*α*_1_	0.091***		0.149***
*β*_1_	0.892***	0.244***	0.276
*β*_2_		0.100***	
λ_0_	0.0003**	-5.647*	2.45E-05
λ_1_	-0.0004***	-1.545	-0.0004***
*τ*			
*μ*			0.032***
*ρ*			0.991***
**diagnostic statistics**			
	*Q*(1) = 0.0004	*Q*(1) = 0.544	*Q*(1) = 0.18
	*Q*(12) = 5.91	*Q*(12) = 5.604	*Q*(12) = 5.71
	*Q*^2^(12) = 20.35	*Q*^2^(12) = 14.74	*Q*^2^(12) = 12.94
	TR^2^(12) = 18.44	TR^2^(12) = 13.48	TR^2^(12) = 12.53
Wald test	10.39***		

Note: *δ* tests the validity of [4] hypothesis, where a positive *δ* indicates that inflation uncertainty increases inflation. λ_0_ is the one-period lagged inflation and tests the validity of [2]–[3] hypothesis, where a positive λ_0_ means that inflation raises inflation uncertainty. Wald test examines the significance of the interactive regime dummy, i.e., λ_1_ = λ_2_ = 0. Rejection of the null hypothesis at the 10%, 5% and 1% significance level are given by the symbols *, ** and ***, respectively.

**Table 10:** GARCH-M models for South Africa.

Coefficients	GARCH-M	EGARCH-M	CGARCH-M
**Conditional mean**			
APART	0.003***	0.004***	0.003***
$\delta_{1}^{SA}$	0.305***	0.348***	0.300***
$\delta_{2}^{SA}$	-0.112*	-0.104	-0.107*
$\delta_{3}^{SA}$	0.042	0.003	0.032
$\delta_{4}^{SA}$	0.131**	0.165***	0.135**
$\delta_{5}^{SA}$	0.129***	0.159***	0.115***
$\delta_{6}^{SA}$	0.001	-0.009	0.005
$\delta_{7}^{SA}$	0.077**	0.064*	0.079**
$\delta_{8}^{SA}$	0.037	0.03	0.036
$\delta_{9}^{SA}$	-0.166***	-0.147***	-0.160***
*δ*	1.557***	1.467***	1.546***
**conditional variance**			
*ϕ*	5.89E-6**	-7.736**	7.14E-06
*α*_1_	0.038		0.001***
*β*_1_	0.135	0.034	-0.001***
*β*_2_		0.158	
λ_0_	0.001***	31.769	-0.073
λ_1_	-0.001**	-70.414**	-0.131
*τ*			
*μ*			0.028**
*ρ*			0.870***
**diagnostic statistics**			
	*Q*(1) = 1.02	*Q*(1) = 1.25	*Q*(1) = 1.27
	*Q*(12) = 13.00	*Q*(12) = 15.21	*Q*(12) = 14.06
	*Q*^2^(12) = 25.96	*Q*^2^(4) = 20.27	*Q*^2^(12) = 25.53
	TR^2^(12) = 21.50	TR^2^(12) = 18.87*	TR^2^(12) = 21.16*
Wald test	10.82***		

Note: *δ* tests the validity of [4] hypothesis, where a positive *δ* indicates that inflation uncertainty increases inflation. λ_0_ is the one-period lagged inflation and tests the validity of [2]–[3] hypothesis, where a positive λ_0_ means that inflation raises inflation uncertainty. APART is a constant dummy variable capturing the effect of apartheid on average inflation. Wald test examines the significance of the interactive regime dummy, i.e., λ_1_ = λ_2_ = 0. Rejection of the null hypothesis at the 10%, 5% and 1% significance level are given by the symbols *, ** and ***, respectively.

**Table 11:** GARCH-M models for Brazil.

Coefficients	GARCH-M	EGARCH-M	CGARCH-M
**Conditional mean**			
APART	0.003***	0.004***	0.003***
$\delta_{1}^{BR}$	0.223***	0.848***	0.227***
$\delta_{2}^{BR}$	-0.294***	-0.228***	-0.031***
$\delta_{3}^{BR}$	0.532***	0.344***	0.777***
$\delta_{4}^{BR}$	0.321***	0.228***	-0.192***
$\delta_{5}^{BR}$	0.069***	0.093***	-0.042***
*δ*	0.063***	0.456***	0.464***
**conditional variance**			
*ϕ*	0.006***	5.321***	0.004***
*α*_1_	6.861***		0.174***
*β*_1_	0.142***	-0.041	0.167***
*β*_2_		0.225***	
λ_0_	0.256***	9.919***	0.054***
λ_1_	-0.427***	-100.193***	-0.080***
*τ*			
*μ*			0.169***
*ρ*			0.812***
**diagnostic statistics**			
	*Q*(1) = 0.593	*Q*(1) = 6.18	*Q*(1) = 1.577
	*Q*(12) = 13.267	*Q*(12) = 46.93	*Q*(12) = 202.72
	*Q*^2^(12) = 0.453	*Q*^2^(12) = 0.135	*Q*^2^(12) = 1.72
	TR^2^(12) = 0.432	TR^2^(12) = 1.51	TR^2^(12) = 1.65
Wald test	1163.90***		

Note: *δ* tests the validity of [4] hypothesis, where a positive *δ* indicates that inflation uncertainty increases inflation. λ_0_ is the one-period lagged inflation and tests the validity of [2]–[3] hypothesis, where a positive λ_0_ means that inflation raises inflation uncertainty. Wald test examines the significance of the interactive regime dummy, i.e., λ_1_ = λ_2_ = 0. The sum of *α*_1_ and *β*_1_ in the conditional variance is larger than one. This is because Brazil was subject to hyperinflation during 1980s to March 1994. Rejection of the null hypothesis at the 1% significance level is given by the symbol ***.

**Table 12:** GARCH-M models for Poland.

Coefficients	GARCH-M	EGARCH-M	CGARCH-M
**Conditional mean**			
D_*t*_	-0.001*	-0.004***	
$\delta_{1}^{PO}$	0.141*	0.272***	0.142*
$\delta_{2}^{PO}$	0.185***	0.264***	0.192***
$\delta_{3}^{PO}$	0.182***	0.114**	0.054
$\delta_{4}^{PO}$	0.001	-7.51E-05	0.026
$\delta_{5}^{PO}$	-0.012	-0.061**	-0.12
$\delta_{6}^{PO}$	0.072***	0.064**	0.068
$\delta_{7}^{PO}$	0.071**	0.081***	0.133***
*δ*	1.439***	1.690***	1.722***
**conditional variance**			
*ϕ*	2.14E-06**	-3.227***	6.15E-06***
*α*_1_	0.024		0.047***
*β*_1_	0.693***	0.035	0.004
*β*_2_		0.017	
λ_0_	0.001***	29.109***	0.001***
λ_1_	-0.001***	-19.046**	-0.001***
*τ*			
*μ*			0.052***
*ρ*			0.562***
**diagnostic statistics**			
	*Q*(1) = 0.01	*Q*(1) = 0.002	*Q*(1) = 13.15
	*Q*(12) = 9.38	*Q*(12) = 2.89	*Q*(12) = 60.50
	*Q*^2^(12) = 27.85	*Q*^2^(12) = 59.30	*Q*^2^(12) = 69.59
	TR^2^(12) = 26.21***	TR^2^(12) = 53.59***	TR^2^(12) = 56.38***
Wald test	12.46***		

Note: *δ* tests the validity of [4] hypothesis, where a positive *δ* indicates that inflation uncertainty increases inflation. λ_0_ is the one-period lagged inflation and tests the validity of [2]–[3] hypothesis, where a positive λ_0_ means that inflation raises inflation uncertainty. D_*t*_ is the monetary regime-constant dummy variable. Wald test examines the significance of the interactive regime dummy, i.e., λ_1_ = λ_2_ = 0. Rejection of the null hypothesis at the 10%, 5% and 1% significance level are given by the symbols *, ** and ***, respectively.

The parameter estimates of the inflation uncertainty proxy, incorporated in the mean equation, have a significantly positive sign for all the countries, indicating that inflation uncertainty increases inflation, as argued by C-M. On the other hand, we find support for the F-B hypothesis; inflation does generate inflation uncertainty, irrespective of the regime followed. Remarkably, the coefficient of the inflation regime slope dummy, λ_1_, employed in the variance equation, is significant and negative in all the respective countries; however, the magnitude of the effect is negligible for Egypt. Note that this ignores the models estimated for Egypt and Jordan with slope political dummy and regime constant dummy. Nevertheless, this result shows that both regimes could to a certain extent reduce inflation uncertainty, albeit not directly, via decreasing the mean of inflation for FER targeters.

For Jordan, the results imply that both negative and positive inflation shocks have the same influence on inflation uncertainty. The positive and significant *β*_2_ for Egypt, presented in the second column, EGARCH, of Table 8, indicates that positive shocks trigger more conditional inflation uncertainty than negative shocks. Similarly, for Brazil, the asymmetric coefficient, presented in Table 11, suggests that inflation uncertainty increases following a positive inflation shock. However, no asymmetry is found for the two other ITers, implying that the inflation uncertainty process is not influenced by the direction of inflation shocks. Interestingly, the slope regime dummy, λ_1_, incorporated in the mean equation, remains negative for all the countries after controlling for asymmetries. Even when CGARCH-M is used in modelling the inflation variance, the slope dummy has a significantly negative sign for all the countries, except for South Africa, where the dummy turns insignificant. This finding suggests that FER system and IT alike are effective in reducing inflation uncertainty in the long run. Generally, the inflation trend of the ITers approaches the mean quicker than that of the FER targeting countries. The power of the short-run component of inflation uncertainty is also higher in the FER targeters. The diagnostic statistics, reported below for each estimated GARCH model, from Tables 6 to 12, indicate that the GARCH models are well-specified. The 1^*st*^ and 12^*th*^ lag order of Ljung-Box and the 12^*th*^ lag squared residuals, as well as the LM test for ARCH, suggest neither remaining autocorrelation nor a non-constant variance for all the countries, except for Poland, where ARCH effect remains in the error terms. Poland adopted different monetary regimes during the 1990s. Moreover, [65] find that the Polish inflation rates are co-moved with that of the Euro Zone. When the sample is split to cover the time period after IT, all GARCH models exhibit no remaining ARCH effect.

The findings of a positive bi-directional relationship between inflation and inflation uncertainty suggest the need for a monetary anchor to reduce both inflation and inflation uncertainty. In general, inflation targeting countries enjoy lower average inflation and inflation persistence compared to the time before adopting IT and compared to the countries with FER regimes. The two monetary regimes appear effective in reducing inflation uncertainty, even under the presence of asymmetries in some cases, like Brazil and Egypt. The effect also remains in the long run, except for South Africa.

For FER countries, the benefits of the regime are not reflected in lower average inflation and inflation inertia. The constant regime dummy variable, incorporated in the mean equation, is found to be insignificant for Jordan and positive for Egypt, and although the FER system appears effective in reducing inflation uncertainty, the magnitude of the regime dummy coefficient is close to zero for Egypt. It could be argued that anchoring the exchange rate could still influence inflation uncertainty as long as the possibility to renege on FER commitment is not perceived by the market. Several depreciations in the Egyptian Pound affected the mechanism of the FER system and its credibility in the market. Weak economic institutions and dependency on political authority due to the absence of mutual and clear division between the central bank and government might hamper the role of the FER as a device for decreasing inflation and inflation uncertainty. It could be argued that providing the market with a quantitative target for the price stability objective would not be optimal if it were not accompanied with a clear framework for the central bank’s roles and objectives, the features which distinguish the IT framework.

The results provide evidence, represented by lower inflation and inflation persistence for inflation targeting countries, that the IT framework which has a direct quantitative target of inflation could be a better signalling device than the soft peg. The institutional features which accompany adopting IT might help the economy to move from high inflation to low inflation levels, as the credibility of the system, and thereby the policy outcome, hinges upon the development of monetary constitutions and transparent policies. The differences in such institutional arrangements might explain why the advantages of the monetary framework differ across countries.

## Conclusions

The current paper examines the relationship between inflation and inflation uncertainty in inflation targeting and exchange rate targeting countries and highlights the role of each monetary anchor in reducing inflation uncertainty. In particular, the paper attempts to underline the impact of monetary policy regimes on inflation and inflation uncertainty using GARCH models over the span 01:1980-06:2014.

The results from the OLS of the conditional mean of inflation, run to the time before and after adopting the examined regime, reveal that inflation targeters, unlike fixed exchange rate countries, have experienced stable inflation after IT. This is confirmed by the statistics of average inflation, in which the ITers have enjoyed lower average inflation after shifting to IT; such desirable effects are not found for exchange rate targeters. This highlights the importance for our target countries, and in general for structurally similar countries, to move to IT if the target is to reduce inflation and inflation volatility.

According to our results, inflation targeting appears to be more effective in lowering inflation persistence and inflation uncertainty than the soft fixed exchange rate regime. However, it is also shown that ITers have not equally benefited from IT. One possible explanation for this might be institutional differences among countries in terms of the level of central bank independence and transparency. Note that countries under the fixed exchange rate lose monetary freedom, as they have to keep their monetary policies in tune with the base country, but they can still ensure institutional independence from the political authorities.

In general, the study has made a way towards enhancing the understanding of the effectiveness of announcing an explicit quantitative target on inflation uncertainty. Furthermore, the positive relationship found between inflation and inflation uncertainty in both directions underlines that a monetary regime with an ultimate goal of price stability is a necessity to keep inflation and inflation uncertainty constrained. Additionally, our findings add to the growing body of literature on the importance of inflation targeting as a framework for monetary policy. IT, according to our results, appears effective in lowering the inflation persistence and inflation uncertainty more than the soft fixed exchange rate regime. However, it is shown that ITers have not equally benefited from IT. One possible explanation for this might be due to the institutional differences among countries in terms of the level of central bank independence and transparency. Therefore, for further contributions, it would be of important interest to analyse the plausible impacts of independent monetary practices and constitutions on inflation and inflation uncertainty, under the two regimes, since exchange rate fixers usually enjoy less institutional and economic independence.

The countries ranked higher based upon the institutional criteria appear to benefit more from the quantitative target policy. This stresses that announcing a quantitative target without a strong, stable and reliable central bank constitution and deeds does not guarantee achieving low inflation and inflation uncertainty. Given this, the preconditions for adopting IT may play a central role in maximising the benefits of adopting a quantitative monetary policy which have important effects on economic welfare.
